# “Grain for Green” driven land use change and carbon sequestration on the Loess Plateau, China

**DOI:** 10.1038/srep07039

**Published:** 2014-11-13

**Authors:** Lei Deng, Zhou-ping Shangguan, Sandra Sweeney

**Affiliations:** 1 State Key Laboratory of Soil Erosion and Dryland Farming on the Loess Plateau, Northwest A&F University, Yangling, Shaanxi 712100, China; 2 Institute of Soil and Water Conservation, Chinese Academy of Sciences and Ministry of Water Resources, Yangling, Shaanxi 712100, China; 3 Institute of Environmental Sciences, University of the Bosphorus, Istanbul, Turkey

## Abstract

Land-use change is widely considered to be a major factor affecting soil carbon (C) sequestration (ΔC*_s_*). This paper studied changes to soil C stocks (C*_s_*) following the conversion of farmland to forest, shrub and grassland across the key area for implementing China's “Grain for Green” — the Loess Plateau. The results are based on a synthesis of 44 recent publications (including 424 observations at 70 sites) which has allowed us to further refine our understanding of the mechanisms driving the increase in C*_s_* following farmland conversion. This synthesis suggests that the ΔC*_s_* potential of the Loess Plateau could reach 0.59 Tg yr^−1^ based on an estimated annual average ΔC*_s_* rate of 0.29 Mg ha^−1^ yr^−1^. In the region's different rainfall zones both the main contributing factors and C*_s_* dynamics varied. Across the entire Loess Plateau, C*_s_* showed first an increasing (<5 yr) then a decreasing (6–10 yr) tendency only to increase (>10 yr) yet again. In addition, the ΔC*_s_* rates depended primarily on restoration age. This synthesis demonstrates that both the initial s C*_s_* and the average annual temperature have a significant effect on ΔC*_s_* while the effect of land-use conversion type, rainfall zone, and average annual precipitation were minimal.

Land-use change significantly impacts the global carbon (C) cycle by changing the rates of both vegetation biomass accumulation and soil erosion^1^,^2^. In the past two centuries, heightened global increases in the conversion of natural vegetation to farmland has led to a net C loss from the terrestrial biosphere into the atmosphere, making this process one of the principle contributors to enhanced global warming^3^. In light of the attempts to reduce atmospheric C, a necessity if we are to come to grips with global climate change, restoring degraded ecosystems has begun to play a significant role in attempts to sequester carbon as a climate change mitigation strategy^4^,^5^,^6^,^7^,^8^. Capturing carbon through changes in land use and sequestering it in the soil is a key component of the “Grain for Green” program.

In the latter half of the twentieth century and increasingly toward its close, sustainable socioeconomic development in China was hindered by widespread environmental degradation. To counteract soil erosion and other environmental problems, in 1999, the Chinese government implemented the “Grain for Green” program to convert farmland to forest, shrub, and grassland^9^. Although the initial goal of the “Grain for Green” program was to control soil erosion on the Loess Plateau, it has been instrumental in increasing both the rate and overall quantity of C sequestered in the soil^7^,^10^. At present, the “Grain for Green” program is the first and still the most ambitious, ecosystem services program in China^6^,^11^,^12^.

The effect the “Grain for Green” program has had on both the accumulation of soil C stocks (C*_s_*) and the rate of soil C sequestration (ΔC*_s_*) has received increasing attention from academics^6^,^7^,^10^,^12^. Zhang *et al.*^12^ reported an average ΔC*_s_* rate of 0.37 Mg ha^−1^ yr^−1^ in the top 20 cm of the soil profile throughout the entire GFG program zone. Four years later, Deng *et al.*^7^ lowered that rate to 0.33 Mg ha^−1^ yr^−1^. Meanwhile, Chang *et al.*^10^ published data suggesting that C*_s_* in the top 20 cm of the soil layer had been accumulating at a rate of 0.712 Tg yr^−1^ (0.173 Mg ha^−1^ yr^−1^) for 60 years across the entire Loess Plateau. Soon after, Feng *et al.*^6^ reported an increase of 2.64 Pg in 2000 to 2.68 Pg in 2008, a ΔC*_s_* rate of 0.085 Mg ha^−1^ yr^−1^ for the uppermost 20 cm of the soil profile from across the plateau. According to the published data, the ΔC*_s_* rate estimated by Chang *et al.*^10^ was roughly double the value reported by Feng *et al.*^6^. This inconsistency begs the question: why is there such a wide range in the reported values? This synthesis sheds light on the contributing factors and clarifies their impact and a new model or estimation/calculation that is more robust. Now is the time to do that.

Researchers face serious challenges in their attempt to accurately estimate changes to the C*_s_* within the area covered by the GFG program^13^. A fundamental drawback in the calculation has been the use of ΔC*_s_* rates following farmland conversion from outside China that may not fit either the climatic or the soil conditions^13^ of the Loess Plateau A second challenge is the small number of actual observations used to estimate the large scale changes in SOC attributed to the GFG program^10^,^12^. Using the CENTURY model to estimate soil carbon storage^6^ in the diverse ecosystems that lay within the area covered by the GFG program led to inaccuracies because the model assumes grassland to be the baseline ecosystem established by restoration and meant to progress through a process of ecosystem amelioration. Some of the results have shown that the model achieves higher results when simulating either farmland or grassland ecosystems than it does for forest ecosystems. Consequently, a more accurate method is required when estimating the rate of change in SOC on such a vast scale.

The Loess Plateau in China, an area of 6.4 × 10^5^ km^2^, is considered one of the most severely eroded areas in the world^14^, making it the main area of interest for implementing China's “Grain for Green” program. Its primary goal was to convert the approximately 2.03 × 10^6^ ha of farmland found on slopes greater than 15° into woodland and grassland^10^,^15^. The effect on C*_s_* or changes to the rate of ΔC*_s_* on the Loess Plateau has been studied since the onset of the program^5^,^16^,^17^,^18^,^19^,^20^. However, because most of the studies were conducted at local sites those processes controlling regional ΔC*_s_* rates remain open to interpretation. In addition, although several authors have analyzed the factors determining C*_s_* during the establishment of perennial vegetation, a consensus on the relative significance of these factors has yet to be achieved^6^,^7^,^10^,^12^, indicating a need for further study on the effects of land-use conversion on ΔC*_s_* rates across the “Grain for Green” zone.

This synthesis of the literature has a three-fold objective: (1) to quantify the potential rate of ΔC*_s_* attributable to the “Grain for Green” or three land-use conversion types (forest, shrub, and grassland) across the Loess Plateau; (2) to assess the effects of land-use conversion on C*_s_* dynamics across the entire Loess Plateau and the region's three rainfall zones (<450, 450–550, and >550 mm); and (3) to determine the extent to which differences in ΔC*_s_* are dependent on land-use conversion types. To achieve these objectives we synthesized the findings of 44 recent publications from the literature in which land use conversion (cropland to forest, shrub and grassland) was related to changes in soil C values on the Loess Plateau.

## Results

**Figure 1:**
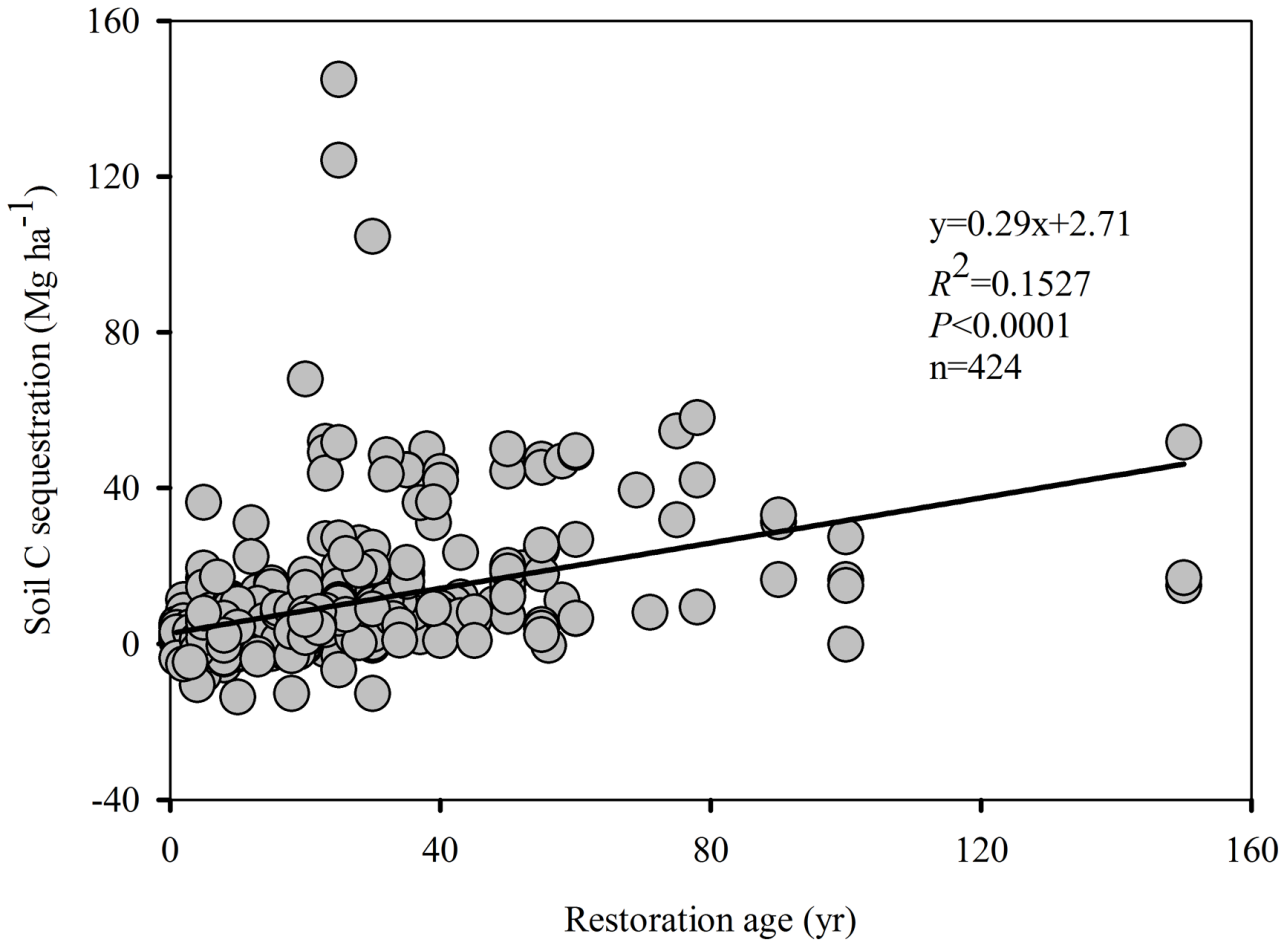
The linear regression equation (y = *k*x + y_0_) between soil C sequestration and restoration age throughout the entire “Grain for Green” program area on the Loess Plateau.

The results show that the relationship between and restoration age was ΔC*_s_* = 0.29 × ΔAge + 2.71 (*R*^2^ = 1.1527, *P*<0.0001) throughout the entire “Grain for Green” program area on the Loess Plateau (Figure 1), providing an estimated average ΔC*_s_* rate of 0.29 Mg ha^−1^ yr^−1 ^(Table 1). The ΔC*_s_* potential of the “Grain for Green” program for the entire Loess Plateau is 0.59 Tg yr^−1^ (Table 1).

**Figure 2:**
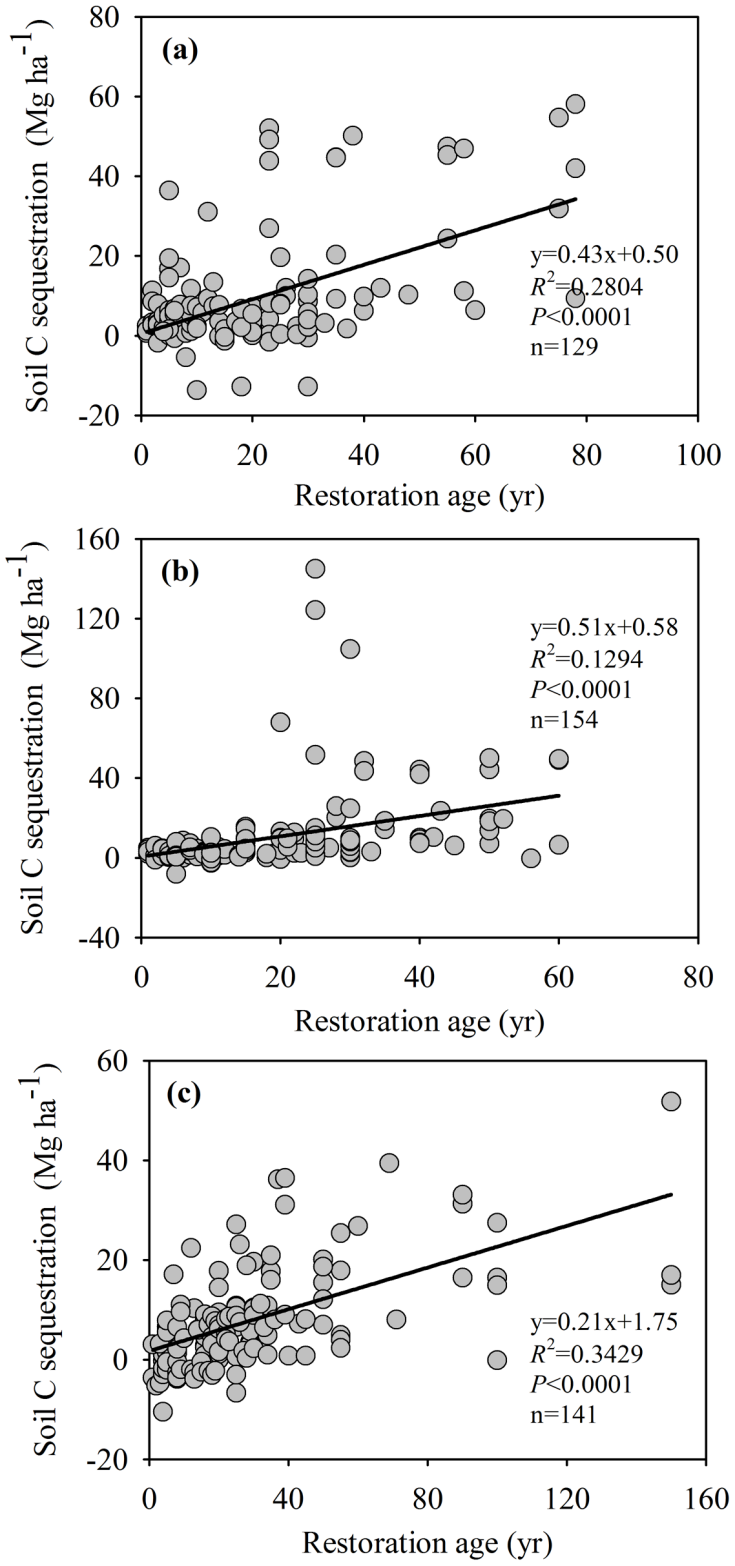
Soil C sequestration rates in different rainfall zones throughout the entire “Grain for Green” program on the Loess Plateau. Note: a. <450 mm; b. 450–500 mm; c. >500 mm.

**Figure 3:**
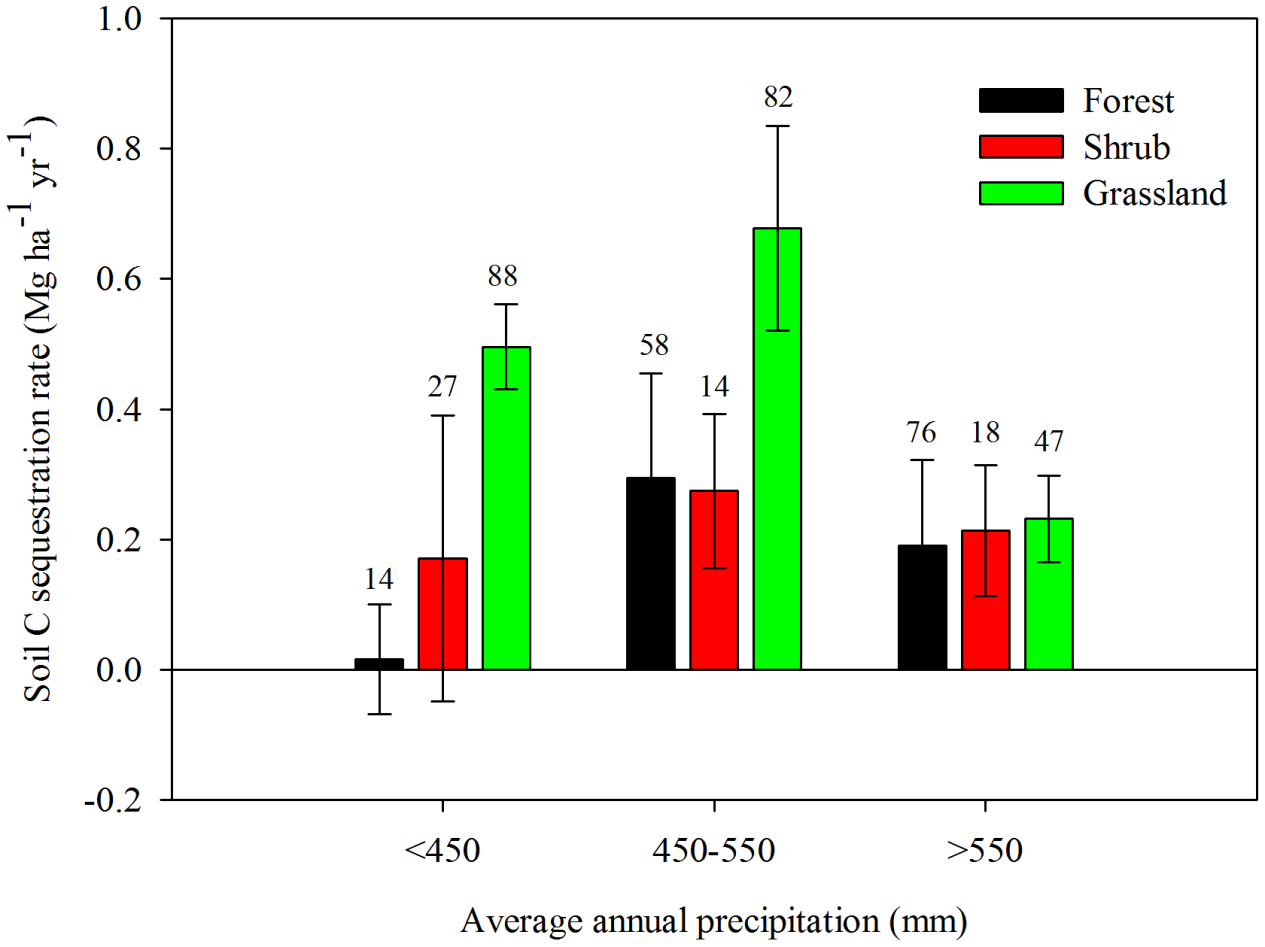
Soil C sequestration rates of different land-use conversion types in the different rainfall zones of the Loess Plateau. Note: The error bars represent standard errors for the slope of Equation 5 (*k*) and values above the bars are the corresponding number of observations.

**Figure 4:**
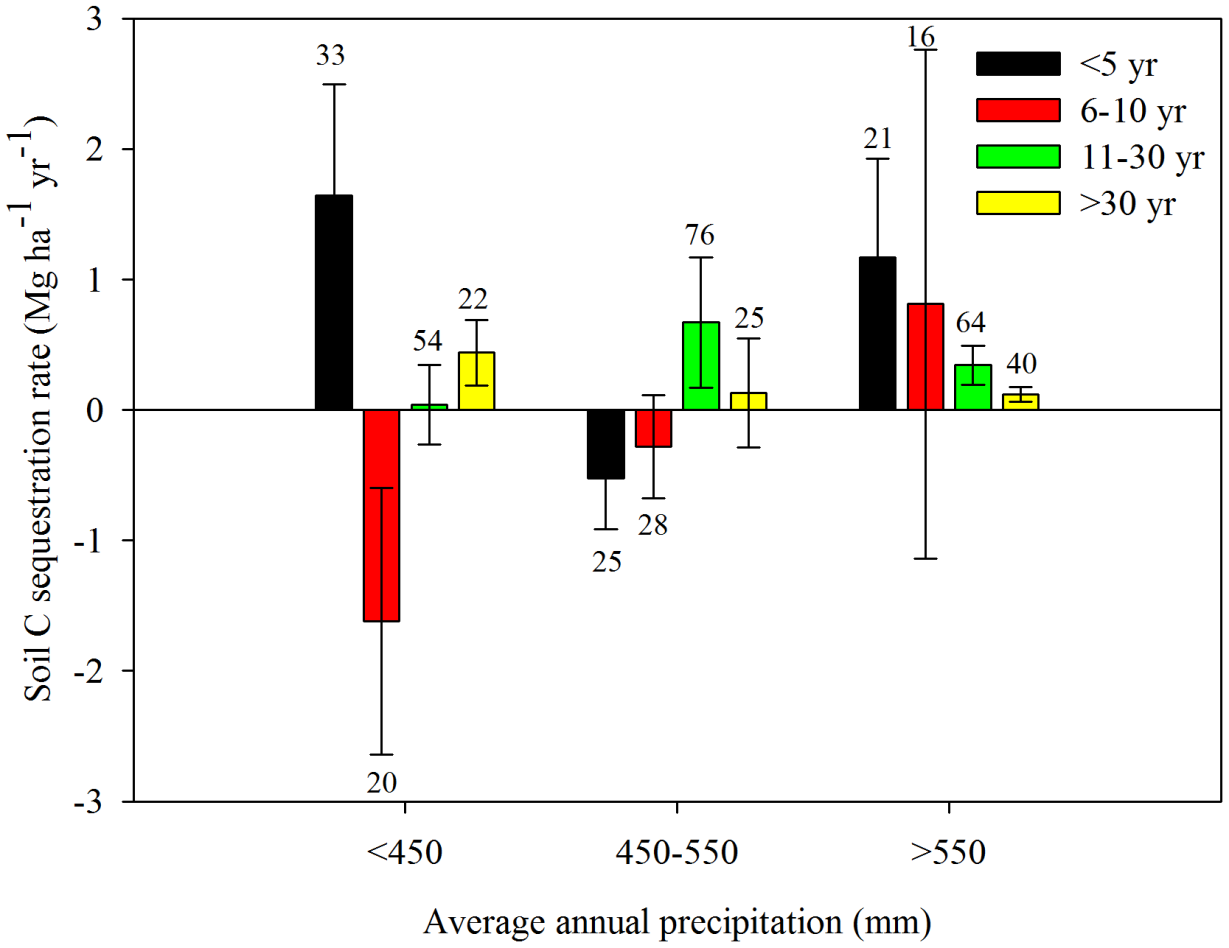
Soil C sequestration rates of different age groups in different rainfall zones on the Loess Plateau. Note: The error bars represent standard errors for the slope of Equation 5 (*k*) and values above the bars are the corresponding number of observations.

**Figure 5:**
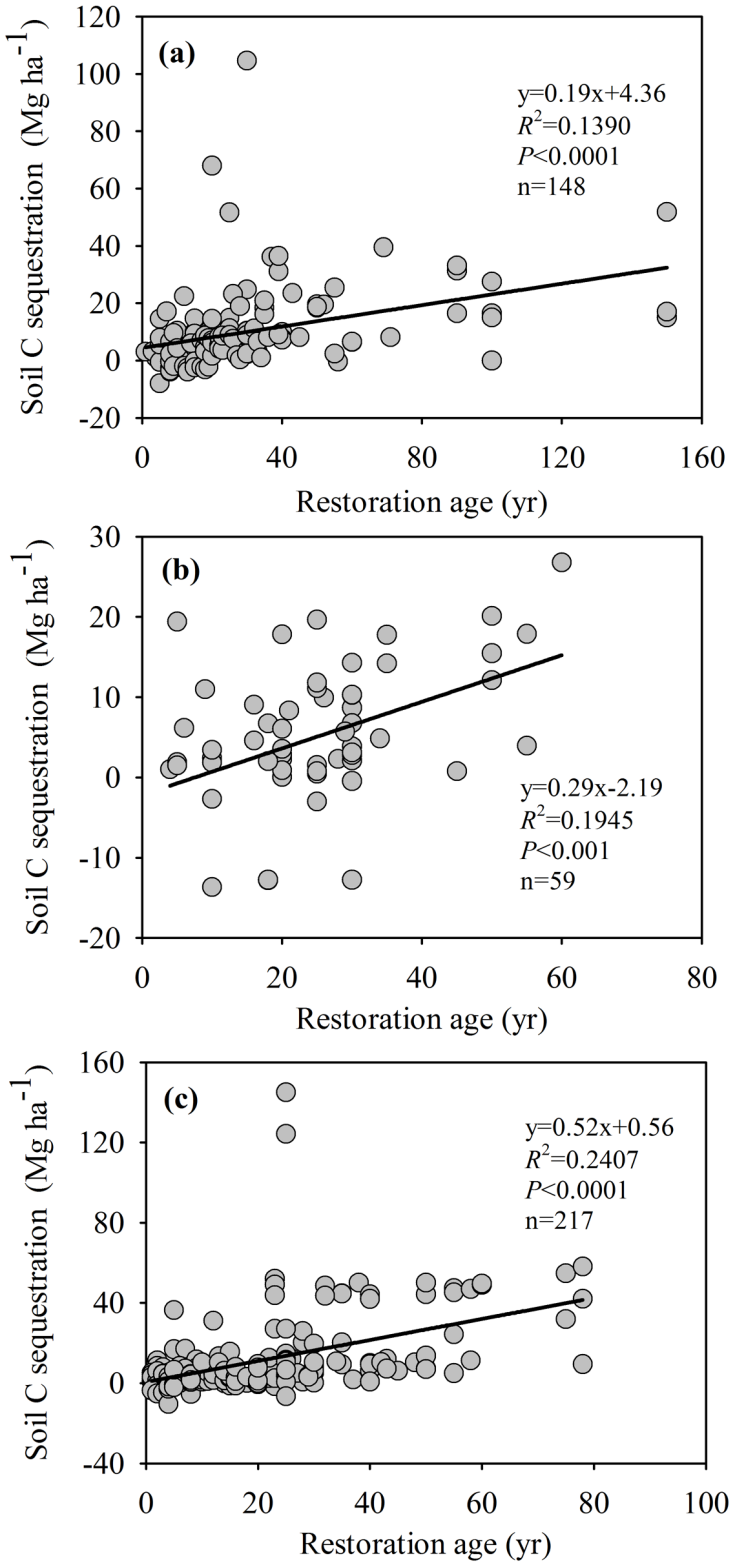
Soil C sequestration rates of different land-use conversion types throughout the entire GFG program area on the Loess Plateau. Note: a. forest; b. shrub; c. grassland.

The three rainfall zones, with annual precipitation averages of <450 mm, 450–550 mm, and >550 mm, had different ΔC*_s_* rates. The 450–550 mm zone had the highest rate at 0.51 Mg ha^−1^ yr^−1^, and the highest precipitation zone (>550 mm) had the lowest rate at 0.21 Mg ha^−1^ yr^−1^ (Figure 2). The dynamics of C*_s_* across the entire plateau was similar to the P<450 mm zone (Figures 1 and 2). During the periods <5, 6–10, 11–30, and>30 years, the respective rates of soil C change in the 0–20 cm soil layer were 0.56, −0.69, 0.45, and 0.11 Mg ha^−1^ yr^−1^ across the entire Loess Plateau. Land converted to grassland had a higher ΔC*_s_* rate than land converted to either forest or shrubland. Both forest and shrubland share similar rates although forest has the lowest rate on the whole (Figure 3). Moreover, the rate for shrubland (0.29 Mg ha^−1^ yr^−1^) was closer to the average level of the whole GFG program zone (Figure 3). In addition, in the different rainfall zones C*_s_* dynamics varied in (1) first increasing (<5 yr) then decreasing (6–10 yr) only to increase (>10 yr) again (<450 mm), and during the periods <5, 6–10, 11–30, and >30 years, the rates of soil C change were 1.65, −1.62, 0.04, and 0.44 Mg ha^−1^ yr^−1^ for 0–20 cm soil, respectively; (2) initial decreases (<10 yr) were followed by a consistent increase (>10 yr) (450–550 mm), and during the periods <5, 6–10, 11–30, and>30 years, the rates of soil C change were −0.53, −0.28, 0.67, and 0.13 Mg ha^−1^ yr^−1^ for 0–20 cm soil, respectively; (3) increasing continuously (0 to >40 yr) (>550 mm), and during the periods <5, 6–10, 11–30, and>30 years, the rates of soil C change were 1.17, 0.81, 0.34, and 0.12 Mg ha^−1^ yr^−1^ for 0–20 cm soil, respectively (Figure 4). We estimate the average ΔC*_s_* rates for the three land-use conversion types (forest, shrub, and grassland) to be 0.19, 0.29 and 0.52 Mg ha^−1^ yr^−1^, respectively (Figure 5).

ANOVA analysis showed that while ΔC*_s_* values indicated no significant difference when related to land-use conversion type or rainfall zone (*P*>0.05), there was a significant difference when measured against restoration age (*P*<0.01) (Table 2). However, in different rainfall zones the main contributing factor varied. In the P<450 mm zone, average annual temperature (T) and restoration age were the main factors while restoration age and initial C*_s_* (I) were the main factors in the P = 450–550 mm zone. Importantly, from a planning perspective, the P>550 mm zone was dominated by one factor alone, the age of restoration. For the whole Loess Plateau, average annual temperature (T) and the age of the restoration were the main factors (Table 3).

## Discussions

### Soil C Sequestration Potential and Dynamics

The global average ΔC*_s_* rates following the conversion of cultivated land to forest, shrub and grassland are 0.45, 0.47 and 1.1 Mg ha^−1^ yr^−1^, respectively^21^,^22^,^23^,^24^. According to our estimates, the average ΔC*_s_* rates in the three land-use conversion types on the Loess Plateau hover around half that of the global average (Figure 5). Across China, average gains of 0.37^12^ or 0.33^7^ Mg ha^−1^ yr^−1^ in SOC following the establishment of perennial vegetation on previously cultivated land have both been reported. Our study estimated the average ΔC*_s_* rate on the Loess Plateau to have been 0.29 Mg ha^−1^ yr^−1^ after farmland conversion (Table 1, Figure 1), a figure which is lower than the average rate for China as a whole^7^,^12^. The most likely reason the ΔC*_s_* rate on the Loess Plateau is lower than both the global and national (China) averages may be that the Loess Plateau is located in the arid and sub-arid zones and consequently experiences lower annual rainfall. We can say this with some conviction because the rate of ΔC*_s_* is positive related with average annual precipitation on a larger scale^7^,^12^,^21^,^22^,^24^. Feng *et al.*^6^ have reported that C*_s_* in the top 20 cm of the soil profile for the entire Loess Plateau increased from 2.64 Pg in 2000 to 2.68 Pg in 2008, a rate of 0.085 Mg ha^−1^ yr^−1^, which is a fraction of the 0.29 Mg ha^−1^ yr^−1^ rate this study reveals. Chang *et al.*^10^ estimated that the C*_s_* in the top 20 cm of the soil profile increased at a rate of 0.712 Tg yr^−1^ over a period of 60 years, a value 13% higher than our results show (0.59 Tg yr^−1^) (Table 1). The differences appear to be attributable to different methods of estimation. Further afield in Central America, where the average annual precipitation is 800 mm, Martens *et al.*^25^ found that ΔC*_s_* grew at an average rate of 0.62 and 1.60 Mg ha^−1^ yr^−1^ following farmland conversion to pasture and secondary forest, respectively. Silver *et al.*^26^ reported that in the top 25 cm soil layer of abandoned tropical agricultural land ΔC*_s_* increased at a rate of 0.41 Mg ha^−1^ yr^−1^ over a 100-year period following afforestation. Those regions have higher ΔC*_s_* rates than areas on the Loess Plateau where the average annual precipitation is ~500 mm, again suggesting that the main reason behind the differing rates of C sequestration might be attributable to differences in average annual precipitation.

**Figure 6:**
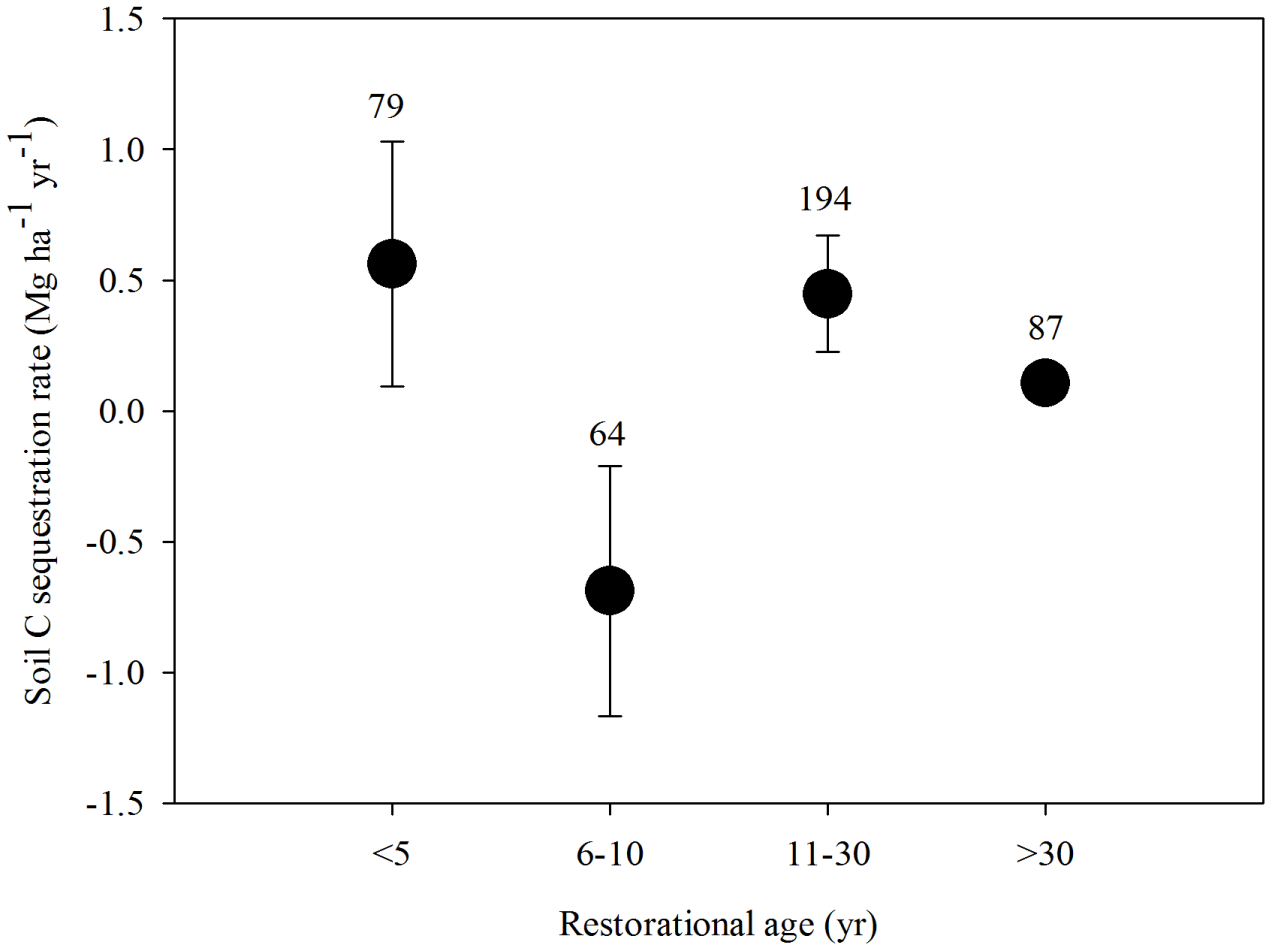
Soil C sequestration rates of different age groups throughout the entire “Grain for Green” program area on the Loess Plateau. Note: The error bars represent standard errors for the slope of Equation 5 (*k*) and values above the bars are the corresponding number of observations.

In our study, C*_s_* in different rainfall zones had varied dynamics, that is: Zone 1: first increased then decreased and then increased again (<450 mm); Zone 2: first decreased and then continued to increase (450–550 mm); Zone 3: increased persistently from the outset (>550 mm) (Figure 4). Although the mechanisms controlling the post-conversion C sequestration rate differ for C*_s_*, precipitation probably driving the variation. Four temporal patterns of change to Csfollowing farmland conversion can be discerned in the literature: (1) an initial decrease in soil C during the early stage, followed by a gradual return of C stocks to farmland level and then an increase to net C gains^12^,^20^,^27^; (2) a decrease^28^; (3) an increase^5^,^29^; (4) unchanged^30^. However, in our study, the Csfirst increased (<5 yr) then decreased (6–10 yr) and then increased (>10 yr) again across the whole Loess Plateau (Figure 6), a finding which differs from that of the national scale^7^, which reported that C*_s_* decreased first (<5 yr) and then increased (>5 yr) following farmland conversion. However, they both show that soil C plays a significant role in fixing soil C. In a review study, Paul *et al.*^31^ determined that the duration of the initial decrease in soil C was reported to have lasted from 3–35 years after agricultural abandonment. However, the pattern was unclear because soil from different depths had been mixed together. In addition, there were great differences among the depths in terms of the temporal change in C*_s_* depending on both climatic regime and soil conditions.

### Factors Affecting Soil C Sequestration after Land-Use Change

Land use change is one of the major factors affecting both variation in Csand the global carbon balance^7^,^17^. It has been previously demonstrated that the “Grain for Green” program is an effective large scale ecosystem services program to restore degraded farmlands^6^,^7^,^10^,^12^,^17^. The results of the synthesis indicate that changes in land use increased soil C stocks, especially when farmland was converted to grassland (Figure 3; Figure 5). Fu *et al.*^18^ reported that shrub was responsible for accumulating more C into soil than grassland, but others documented no difference between the two land-use types^32^. When comparing the effects of different land-use conversion types on ΔC*_s_*, Chang *et al.*^10^ reported no difference among grassland, shrubland and forest on the northern Loess Plateau (<450 mm); moreover, soil carbon in forested systems increased much more than in shrubland or grassland on the central Loess Plateau (450–550 mm); on the southern Loess Plateau (>550 mm), forest had a stronger effect on the rate of ΔC*_s_* than grassland, but a non-significant effect for shrubland. However, the results from the synthesis show no significant difference (*P*>0.05) in ΔC*_s_* values for the different land-use conversion types.

The length of time since land-use conversion plays a consistent and key role in estimating soil C stocks^7^,^12^,^24^. This study reveals a significant difference associated with age groups (*P*<0.01) (Table 2) with ΔC*_s_* showing a significant positive correlation with restoration age (Table 4). This is mainly because ΔC*_s_* increased as the quantity of C inputs increased, a process which was accompanied by a new microclimatic regime and enhanced organic matter protection of the soil^33^. However, in the first few years of plantation establishment, a reduction in C*_s_* was frequently observed^12^,^31^, as it was in the 450–500 mm zone of this study. In one case, the C*_s_* decreased dramatically as a result of more serious erosion brought about by a lack of agricultural maintenance practices, fertilization, and sufficient vegetation cover, after which the farmland was soon abandoned (<4 year)^34^.

Deng *et al.*^7^ have reported that ΔC*_s_* shows no significant correlation with either average annual temperature or precipitation at the national (China) scale, yet temperature and precipitation are the main factors determining changes to the rate of Csin the later stage (>30 years) of restoration. Our results show that ΔC*_s_* in the three rainfall zones shows no significant difference on the Loess Plateau (*P*>0.05) (Table 3). It may be because the Loess Plateau is a relatively small region with an almost uniform coverage of low precipitation. Most sites are distributed between 300 and 600 mm (See appendix dataset S1) meaning the effect of rainfall among the three rainfall zones is not statistically significant. Although the correlation between ΔC*_s_* and average annual precipitation was not significant (*P*>0.05) the two features are positively correlated (Table 4) which demonstrates that precipitation increases the magnitude of ΔC*_s_* following land-use conversion. In our study, ΔC*_s_* had a significant negative correlation with average annual temperature (*P*<0.05) (Table 4), this may be because higher temperatures lead to higher losses of soil C through decomposition of soil organic matter. However, Paul *et al.*^31^ had reported that soil C accumulated with increasing mean annual temperature and Deng *et al.* (2014) also found that soil C had a significant positive correlation with mean annual temperature at the national (China) scale, but it was not significant (*P*>0.05). The inconsistency may be due to differences in scale in the study areas.

Moreover, ΔC*_s_* showed a significant positive correlation with initial C*_s_* (*P*<0.05) (Table 4), a finding which contradicts the results of both Zhang *et al.*^12^ and Deng *et al.*^7^. Both studies are focused at the national scale whereas our study focuses on a typical region in China, the Loess plateau. Thus, the discrepancy appears to be attributable to a difference in scale. The difference in climatic conditions caused by the vastly differing scales is the main distinguishing factor in the varying rates of ΔC*_s_* since land use conversion reported in the literature. In addition, our synthesis revealed that initial C*_s_* are strongly correlated with average annual temperature and precipitation (Table 4). As we know, vegetation restoration, either natural or purposeful, depends on recovery based on the condition of the original land. Obviously, basic conditions, such as soil nutrients, water, and climate have a profound effect on the process of vegetation restoration meaning that the rate of soil carbon change is not independent of initial SOC.

### Management Implications

The initial goal of the “Grain for Green” program was to control soil erosion on the Loess Plateau, however, this program has also come to play a significant role in soil carbon sequestration^6^,^7^,^10^,^12^. To facilitate the value of C sequestration and soil conservation while simultaneously mitigating against the threat posed by ever-increasing levels of atmospheric carbon, it is essential to both plan for more land-use conversion and to enhance the quality of those areas which already exist through suitable management regimes. Restoration programs need to be based on the average rate of ΔC*_s_* according to land-use type and the durability of the associated carbon sequestration process. In the lower annual precipitation zone of the Loess Plateau, grassland displayed a higher rate of ΔC*_s_* compared to either forest or shrubland (Figure 3), making grassland the logical choice for these sites (<450 mm). In the central precipitation (450–550 mm) zone, both grassland and forest displayed higher ΔC*_s_* rates when compared to shrub (Figure 3) meaning grassland and forest systems are recommended. In the high precipitation zone (>550 mm), although forest displayed lower ΔC*_s_* rates compared to shrub and grassland, they were basically the same in magnitude (Figure 3), so forest, shrub and grassland can all be justified for this rainfall zone. In addition, across the Loess Plateau the average rate of ΔC*_s_* slows after about 30 years from the time of initial farmland conversion (Figure 6) which indicates a need for careful land-use management practices to maintain optimal levels of soil C stocks. In the P<450 mm zone, the rate of ΔC*_s_* remained at a high level 30 years after conversion. Consequently, to enhance the benefits of ΔC*_s_* priority should be given to long-term enclosure.

### Uncertainity Analysis

This synthesis offers the most accurate estimate on which to base the potential rate of ΔC*_s_* across the entire “Grain for Green” program zone, albeit with one caveat. Strict accuracy is limited due to the uneven distribution of data collected across the Loess Plateau. Some uncertainties derive from the temporal pattern of SOC accumulation, which several studies have reported to be non-linear^5^,^12^. Additionally, many of the studies have no long term observations and consequently, these measurements may add to the uncertainty. In addition, in our study, we ignored the effect of bulk density substituting Equation (2) and Equation (3), i.e., the C_s_ will be equal as long as SOC is the same. In reality, however, bulk density would not only be significantly different among different sites but also experience significant change after land use conversion within a site. In future, we should focus on the effect(s) of the sites and land use change on soil bulk density to build a functional relationship between SOC and soil bulk density.

## Methods

### Data Preparation

**Figure 7:**
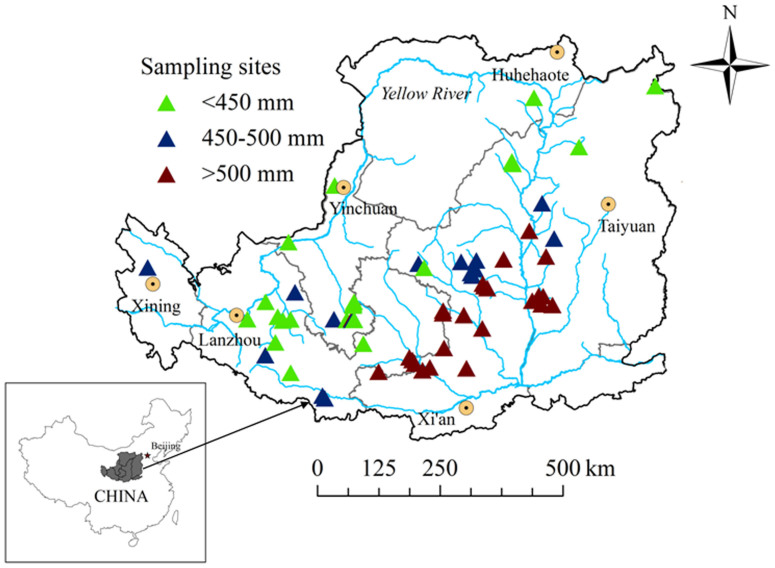
Distribution of “Grain for Green” program sampling sites on the Loess Plateau reported in the collected peer-reviewed papers. Note: the number of sampling sites for the 3 rainfall zones (<450, 450–550, >550 mm of average annual precipitation) were 22, 18 and 30 respectively. The software ArcGis 9.3 was used to create the map[s].

We collected the available published literature (1999–2012) on changes to soil C following the conversion of long-term cultivated farmland to forest, shrub, and grassland as part of the “Grain for Green” program on the Loess Plateau. The raw data were either obtained from tables or extracted by digitizing graphs using Get Data Graph Digitizer (ver. 2.24, Russian Federation). For each paper, the following information was compiled: sources of data, site longitude and latitude, climatic information (average annual temperature and average annual precipitation), land-use conversion type (farmland, forest, shrub, grassland), years since farmland conversion (restoration age), soil depth, experimental design (paired site, chronosequence, retrospective design), soil bulk density, and amount of SOC or ΔC*_s_* in the top 20 cm of the soil profile (Appendix dataset S1). We only estimated ΔC*_s_* for the top 20 cm of the soil because 92% of studies investigating land-use conversion on the Loess Plateau^18^,^35^ found that ΔC*_s_* occurs mainly in the surface soil following land-use conversion from farmland to either natural vegetation or plantation^10^,^17^,^36^. In addition, studies have shown that those significant differences in soil C observed in the topsoil do not extend to the subsoil. Our final dataset was composed of 44 papers encompassing 424 observations in the “Grain for Green” program zone of which 43 papers accounted for 256 observations while the other 168 observations were from Chang *et al.*^10^. The sites reported from the literature are widely distributed across the “Grain for Green” program zone as shown in Figure 7.

The Loess Plateau can be divided into three main rainfall zones: the northern Loess Plateau with precipitation <450 mm, the central Loess Plateau with precipitation between 450 and 550 mm, and the southern Loess Plateau with precipitation >550 mm^37^, allowing us to compare the effects of rainfall on SOC accumulation following land-use conversion. Restoration age was divided into four groups, as follows: 0–5, 6–10, 11–30, and >30 years.

### Data Calculation

Of the literature-collected data, soil carbon stocks expressed in units of “kg m^−2^” were transformed to “Mg ha^−1^”.

If the samples only reported soil organic matter (SOM) content their SOC values were calculated using the relation between SOM and SOC using the following equation^38^: 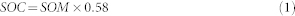
In the synthesized data, not all the sampling data were missing soil bulk density (BD) values. We only used the empirical relationship between soil organic carbon content (C) and bulk density for those results in which soil bulk densities had not been measured in the original papers. We used the empirical relation between soil organic carbon content (SOC) and BD^39^, which was also used by Zhang *et al*.^12^ and Deng *et al*.^7^: 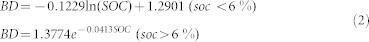
The SOC stock was calculated using the following equation: 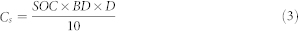
in which, C*_s_* is the soil organic carbon stock (Mg ha^−1^); SOC is soil organic carbon concentration (g kg^−1^); BD is soil bulk density (g cm^−3^); and D is soil thickness (cm).

The ΔC*_s_* rate was estimated depending on changes in ΔC*_s_* at different time intervals. The study set the value of C stocks for farmland as the baseline from which to calculate the ΔC*_s_* rates during the restoration process when farmland is converted into forest, shrub or grassland. We first calculated the amount of sequestered C for each afforested site following farmland conversion, 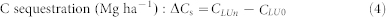
in which, C_LUn_ represents soil C stocks at afforested sites (Mg ha^−1^), and C_LU0_ is the initial soil C stocks (farmland) (Mg ha^−1^).

Secondly, we constructed the linear regression equation (y = f(x) = y_0_ + *k*x) between C sequestration (ΔC*_s_*) and the age for each age group or the whole restoration chronosequence, 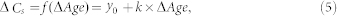
we know that the equation's first derivative represents the rate of change of the curve, so Equation 5's first derivative of ΔC*_s_* versus ΔAge represents the rate of change in the carbon stocks (soil C sequestration rates)^7^: 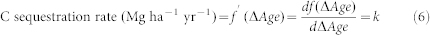
in which, y_0_ is Equation 5's constant; *k* is the rate of change in C stocks (Mg ha^−1^ yr^−1^) and also represents the slope of Equation 5, and ΔAge represents the time interval (year), ΔAge>0.

The ΔC*_s_* potential within the “Grain for Green” program on the Loess Plateau was estimated using the ΔC*_s_* rate and the area of farmland or degraded land in the program. The potential for ΔC*_s_* was estimated using the ΔC*_s_* rate and the area of farmland or degraded land within the program. For our study, we relied upon the now classic description proposed by Chang *et al.*^10^ for whom that area of the Loess Plateau which fell under the “Grain for Green” program was composed of farmland characterized by 15 degrees or more of slope. The areas of farmland, including the criterion of rainfall zones, were obtained by overlaying a land-use map of the entire Loess Plateau in 2000, the launch year of the “Grain for Green” program, with a 90-m resolution digital elevation model (DEM). The land-use map was obtained using Landsat TM and ETM remote sensing in 2000. Land-cover categories, including that of farmland, were divided using the 200 m × 200 m pixel output images. These data were used to estimate the ΔC*_s_* potential of the “Grain for Green” program across the entire Loess Plateau.

### Statistical Analysis

Multi-way ANOVA was performed to test the effects of the difference among land-use conversion types and age groups in the different rainfall zones. Differences were evaluated at the 0.05 significance level. Stepwise regression analysis was used to analyze the relationship between ΔC*_s_* following farmland conversion and average annual temperature (T), average annual precipitation (P), years since farmland conversion (A), and initial C*_s_* in the 0–20 cm (I) of every age group. Pearson correlation analysis was used to study the relationship between ΔC*_s_* following farmland conversion and T, P, A, and I of all data. All statistical analyses were performed using the software program SPSS, ver. 18.0.

## Author Contributions

L.D., Z.S. designed the study, L.D. conducted the study, L.D., Z.S., S.S. wrote the paper, L.D., Z.S., S.S. revised the paper.

## Supplementary Material

Supplementary Information Dataset 1

## Figures and Tables

**Table 1:** 0–20 cm actual and potential soil C sequestration rates for the “Grain for Green” program on the Loess Plateau

Item	C sequestration rate (Mg ha^−1^ yr^−1^)	Land-use conversion	Average restoration age (yr)	Area* (ha)	C sequestration potential (Tg yr^−1^)
Whole GFG	0.29 ± 0.03	Forest, shrub, grassland	23	2.03 × 10^6^	0.59 ± 0.07

Note: * The data has been adopted from Chang et al. (2011). The error bars represent standard errors for the slope of equation 5 (*k*).

**Table 2:** Multi-way ANOVA of soil C sequestration in different rainfall zones, land-use conversion types, and age groups on the Loess Plateau

Source	*df*	F	Sig. (*P*)
Rainfall zones	2	1.644	0.194
Land-use conversion types	2	0.254	0.776
Age groups	3	5.318	0.001**
Rainfall zones × Land-use conversion types	4	2.150	0.074
Rainfall zones × Age groups	6	0.893	0.500
Land-use conversion types × Age groups	6	0.829	0.548
Rainfall zones × Land-use conversion types × Age groups	10	1.365	0.194

Note: **indicates significant difference at the 0.01 level (*P*<0.01).

**Table 3:** Stepwise regression to detect factors (T, P, A and I) determining soil C sequestration following land-use conversion in 0–20 cm soil on the Loess Plateau

Rainfall zone	Equation	*R*^2^	Sig. (*P*)	n
<450 mm	ΔC*_s_* = −5.00T + 0.37A + 36.42	0.491	0.000**	129
450–550 mm	ΔC*_s_* = 0.63A + 0.74I-6.57	0.206	0.000**	154
>550 mm	ΔC*_s_* = 0.21A + 1.70	0.356	0.000**	141
All	ΔC*_s_* = −2.78T + 0.28A + 26.46	0.202	0.000**	424

Note: ΔC*_s_* is soil C sequestration following land-use conversion; T (°C) is the average annual temperature; P (mm) is the average annual precipitation; A (yr) is the restoration age; I (Mg ha^−1^) is the initial soil C stocks.

**Table 4:** Pearson correlation coefficients between soil C sequestration and factors: average annual temperature, average annual precipitation and initial soil C stocks following land-use conversion on the Loess Plateau

	Restoration age (yr)	Average annual temperature (°C)	Average annual precipitation (mm)	Initial soil C stocks (Mg ha^−1^)
Soil C sequestration (Mg ha^−1^)	0.391** (424)	−0.233** (424)	0.017 (424)	0.159* (256)
Initial soil C stocks (Mg ha^−1^)	-	−0.438** (256)	0.210** (256)	-

Note: **Correlation is significant at the 0.01 level (2-tailed) (*P*<0.01), and *Correlation is significant at the 0.05 level (2-tailed) (*P*<0.05); (value) indicates the number of observations.
